# To Erase or Not to Erase: Non-Canonical Catalytic Functions and Non-Catalytic Functions of Members of Histone Lysine Demethylase Families

**DOI:** 10.3390/ijms25136900

**Published:** 2024-06-24

**Authors:** Elena Di Nisio, Valeria Manzini, Valerio Licursi, Rodolfo Negri

**Affiliations:** 1 Department of Biology and Biotechnologies “C. Darwin”, Sapienza University of Rome, 00185 Rome, Italy; elena.dinisio@uniroma1.it (E.D.N.); valeria.manzini@uniroma1.it (V.M.); 2 Institute of Molecular Biology and Pathology (IBPM), National Research Council (CNR) of Italy, 00185 Rome, Italy; valerio.licursi@cnr.it

**Keywords:** histone demethylases, catalytic-independent functions, cancer epigenetics, histone modifications, protein isoforms

## Abstract

Histone lysine demethylases (KDMs) play an essential role in biological processes such as transcription regulation, RNA maturation, transposable element control, and genome damage sensing and repair. In most cases, their action requires catalytic activities, but non-catalytic functions have also been shown in some KDMs. Indeed, some strictly KDM-related proteins and some KDM isoforms do not act as histone demethylase but show other enzymatic activities or relevant non-enzymatic functions in different cell types. Moreover, many studies have reported on functions potentially supported by catalytically dead mutant KDMs. This is probably due to the versatility of the catalytical core, which can adapt to assume different molecular functions, and to the complex multi-domain structure of these proteins which encompasses functional modules for targeting histone modifications, promoting protein–protein interactions, or recognizing nucleic acid structural motifs. This rich modularity and the availability of multiple isoforms in the various classes produced variants with enzymatic functions aside from histone demethylation or variants with non-catalytical functions during the evolution. In this review we will catalog the proteins with null or questionable demethylase activity and predicted or validated inactive isoforms, summarizing what is known about their alternative functions. We will then go through some experimental evidence for the non-catalytical functions of active KDMs.

## 1. Introduction

N-terminal histone tails can be dynamically modified by the addition and/or the removal of post-translational modifications, including methylation [1]. Histone methylation is deeply involved in the control of crucial biological processes such as transcription regulation [2], DNA replication [3], and genomic damage signaling and repair [4,5]. For this reason, it is tightly controlled during cellular processes [6] by the coordinated and balanced action of histone methyltransferases and demethylases [7]. Lysine demethylases (KDMs) include the class of flavine-dependent amine oxidases (also known as the KDM1 subfamily), which act on mono- and di-methylated lysines, and the large class of Jumonji C (JmjC) domain-containing proteins (also known as the KDM2–KDM8 subfamilies), which are Fe(II) and 2-oxoglutarate (2OG)-dependent enzymes [8,9]. All the members of this second group are JmjC domain-sharers but, depending on the degree of homology and the presence of other domains, they can be classified into subfamilies, often sharing common substrates [10]. Lysine demethylation can also occur on non-histone proteins and has been implicated in human tumorigenesis (reviewed in [11]). Here, we will focus on the non-canonical catalytic functions and the catalytic-independent functions of proteins belonging to the lysine demethylase families. Indeed, evolutionary branching has occurred in the history of the 2OG-dependent oxygenases, thereby distinguishing hydroxylases and demethylases with distinct cellular functions [12]. Interestingly, JmjC-only proteins mainly act by modifying other proteins or ssRNA [13], rather than by catalyzing demethylation [8]. JmjC proteins can therefore act as hydroxylases, functioning as oxygen sensors, or perhaps as endopeptidases, to regulate both protein–protein interactions and protein stability, but also transcription-related processes. Besides the catalytic activities different from lysine demethylation, a section of this review will be dedicated to emerging topics related to the demethylase-independent functions of some KDM proteins. We speculate how some KDM transcripts may potentially or effectively give rise to multiple protein isoforms including—or not—JmjN and/or JmjC domains, both of which are required for the catalytic activity of KDM4s and KDM5s, in light of the potential relevance of such isoforms’ role in demethylase-independent functions.

## 2. Different Functions Besides Histone Demethylation Activity

In addition to active histone demethylases, the KDM family includes proteins with no or uncertain histone demethylase activity, such as JARID2 and UTY, and JmjC-only domain proteins with other catalytic functions. Moreover, some catalytically active KDMs possess catalytic-independent functions. These three different groups are listed in Table 1.

### 2.1. Proteins Containing Jumonji Domains But Retaining No or Uncertain Histone Demethylase Activity

#### 2.1.1. JARID2: Inactive But Essential

JARID2 shows high sequence homology with the KDM5 subfamily [8,14] at its C-term portion [60]. Despite the different spatial organization of the conserved domains, they share the Jumonji N (JmjN) domain, the A-T rich interaction domain (ARID), the Jumonji C (JmjC) domain, and the Zinc-finger (zf)-C5HC2 domain [8]. However, they differ in the presence of the Plant homeodomains (PHDs), absent in JARID2 [8], and in the N-terminal portion, which is not shared by the KDM5 members. The Polycomb repressive complex 2 (PRC2) interacting domain, the nucleosome binding domain, and the ubiquitin-interaction motif reside at the N-term region of JARID2 [60,61,62]. Intriguingly, evolutionarily conserved substitutions in crucial catalytic residues of JARID2 impair its demethylase activity [8,63], representing a clue for the presence of important histone demethylation-independent functions. JARID2 is indeed required for the proper differentiation of embryonic stem cells, resulting in an essential component for normal embryonic development [14,60,64,65,66,67]. In keeping with this, JARID2 mutant mice experienced lethality in later embryonic stages [14]. Despite being inactive as histone demethylase, JARID2 influences the distribution of the H3K27me3 mark during developmental and cellular differentiation processes [14,60,63,68,69,70,71,72,73,74] by modulating the chromatin association of PRC2 complex, and potentially its enzymatic activity [61,68,70]. The JARID2-mediated recruitment of PRC2 on target genes can be accomplished by the putative DNA-binding properties of its ARID and zf-C5HC2 domains [70], by its N-terminal region’s ability to interact with nucleosomes [61], by favoring lncRNA-mediated interaction [75,76] or by binding to the H2AK119ub modification, which is deposited by the PRC1 complex [62]. JARID2 thus exerts a crucial role in PRC2 recruitment for gene expression regulation. Accordingly, in lineage-committed human cells, the production of a cleaved ∆N-JARID2 form that lacks the N-terminal PRC2-interacting domain can promote activation switching of differentiation genes. However, the consolidated role of JARID2 in PRC2 recruitment [60,62,68] is not limited to simple targeting. Growing evidence shows that JARID2 might regulate the methyltransferase activity of the PCR2 complex differently depending on the chromatin context [14,61,69,71], also functioning itself as a PRC2 substrate [77] and inducing allosteric activation to increase the catalytic activity of the complex [73]. Beyond the modulation of the H3K27me3 mark, JARID2 can also mediate the distribution of other epigenetic repressive marks, including H3K9me1 and H3K9me2/3, which mediate the recruitment of methyltransferases including G9a–GLP complex and SETDB1 [78,79,80]. In conclusion, although catalytically inactive, JARID2 can target chromatin by exploiting the ARID and the zf-C5HC2 domains or the N-terminal region and exerts a crucial non-catalytical regulatory function on the assembly of repressive complexes and histone modification.

#### 2.1.2. UTY: A Catalytically Inactive KDM6 Subfamily Member?

The KDM6 group includes three members, KDM6A (ubiquitously transcribed tetracopeptide repeat on X chromosome, also known as UTX), KDM6B (or JMJD3), and UTY (ubiquitously transcribed tetracopeptide repeat on Y chromosome, also named KDM6C). Unlike KDM6A and KDM6B, current in vitro and in vivo evidence suggest that UTY possesses null or very low demethylase activity due to sequence variation within its catalytic domain [15,16,17,58]. It nevertheless seems to have relevant functions during development [15]. Homozygous KDM6A-KO mouse females, but not hemizygous KDM6A-KO male embryos expressing normal levels of UTY, exhibit embryonic lethality, suggesting that UTY is functionally compensating for certain aspects necessary for development in males lacking KDM6A [15]. The compensation is not dependent on H3K27 demethylation activity, but, rather, linked to other proteins and protein complexes that broadly regulate transcription [15,81]. Interestingly, the expression patterns of KDM6A and UTY differ greatly in mouse brains, with male mice displaying a much higher expression of UTY than KDM6A in the hypothalamus, suggesting a new role for UTY in neuroendocrine regulation [82]. In adult mice, developmental transcriptomic patterns can be altered by transgenic overexpression of UTY [81]. Specifically, a global UTY expression caused a masculinization in the female gene expression patterns in both the placenta and hypothalamus [81], which respond to changes in the intrauterine environment in a sex-specific manner. However, as the authors admitted, the limitation of such an experiment is the absence of a conditional and tissue-specific expression of the gene that should be performed to rule out indirect effects due to changes in other tissues [81]. Overall, if the extensive sequence homology between UTY and KDM6A may suggest similar and possibly partially redundant functions, some differences, such as the presence of conserved substitutions that abolish the ability of UTY to demethylate the H3K27me2/3 or the different tissue-specific and sex-dependent expression patterns of these two proteins, do suggest the existence of important functions of UTY that are independent on its demethylase activity and not yet fully understood.

### 2.2. The JmjC Domain-Only Family: Enzymes with Unclear Demethylase Activity But Which Show Different Catalytic Functions

The majority of JmjC hydroxylases catalyze the demethylation of histone lysines, playing a role in epigenetics [8]. However, the JmjC-only members retain only the JmjC domain and no other identifiable protein domains possibly involved in chromatin biology [8]. They were initially considered bona fide histone demethylases for the presence of the Jumonji C domain, but they showed questionable histone demethylase activity (Table 1), and, rather, turned out to have different enzymatic activities. It has been proposed that these proteins might have diverged in higher eukaryotes to carry out biological functions independently of histone demethylation [8], but more recent structural analyses surprisingly support the hypothesis that, conversely, KDMs evolved from the JmjC-only hydroxylase family [12].

So far, ten 2OG-dependent oxygenases have been identified as JmjC-only proteins in humans: HIF1AN, HSPBAP1, JMJD4-8, RIOX1, RIOX2, and TYW5 [21]. These enzymes catalyze oxidative reactions such as hydroxylation and possibly demethylation, always through a mechanism of demethylation via a hydroxylation reaction [21]. Some JmjC-only proteins, such as JMJD7, are known to localize to the cytoplasm; others, such as HSPBAP1, at the mitochondria; and others, such as the RIOX1/RIOX2 group, are prevalently nuclear or can shuffle between the cytoplasm and nucleus, as in the case of HIF1AN. These small enzymes exert several functions by hydroxylating proteins and RNA, and their dysregulation has been linked to cancer development (reviewed in [22]).

#### 2.2.1. HSPBAP1

Heat shock-associated protein 1 (HSPBAP1, also known as PASS1 for protein associated with small stress protein 1) was identified as an interactor of the 27kDa heat shock protein (HSP27) which negatively regulates its capacity to protect cells from heat shock [18]. Since its discovery, little has been known about this protein, whose orthologues exist from flies to humans [83]. In Drosophila, it appears to be involved in ethanol tolerance [84]. In humans, it is expressed in a wide variety of tissues, but it has been found abnormally expressed in the anterior temporal neocortex of patients with intractable epilepsy (IE) [85], suggesting its pathogenic role in these patients. Apart from the observed correlation between HSPBAP expression and the associated phenotypes, the significance and the molecular mechanism underlying them are still unclear. It has been indeed hypothesized that the effects of HSPBAP1 depend on its opposite effect on Hsp27. However, besides their molecular interaction, no further investigation has been conducted to clarify the possible involvement of oxidative reactions, such as hydroxylation, as post-translational modifications that might impact Hsp27 stability or activity, as often occurs for other hydroxylases belonging to this group.

#### 2.2.2. RIOX1/RIOX2

Ribosomal oxygenase 1 (RIOX1), also known as Nucleolar protein 66 (NO66), and Ribosomal oxygenase 2 (RIOX2), also known as myc-induced nuclear antigen (MINA), are hydroxylases highly selective for histidine residues [86]. This ribosomal oxygenase group has orthologues from prokaryotes to humans [20,87]. They share a similar protein domain architecture, consisting of a JmjC domain with the evolutionarily conserved aa triad HDH for enzymatic activity, a dimerization domain, and a winged-helix domain (WH) [87]. In addition, RIOX1 possesses an extended N-terminal domain with a nucleolar localization signal [87]. Consistent with their nucleolar subcellular localization [20,87,88,89,90], RIOX1 and RIOX2 interact with the 60S ribosomal proteins RPL8 and RPL27A, respectively, catalyzing their hydroxylation on specific histidine residues [12,20,21,22]. The nucleolar accumulation of RIOX2 seems strictly connected to the cell’s metabolic state, since it occurs mainly under cell proliferation stimuli, in occurrence with active ribosomal gene transcription [90]. Interestingly, c-myc positively regulates both the expression of genes involved in the synthesis and processing of rRNA and ribosomal proteins [91] and RIOX2 expression, but lacks a clear correlation in this case [90]. Nevertheless, RIOX2 knockdown reduces the proliferation of transformed rodent and human cell lines, indicating a potential role in cell proliferation induced by c-myc [88,92], and RIOX2 expression levels are increased as a result of exposure to cancer-associated chemical elements [93,94], suggesting a potential role in cancer onset. In addition to nucleolar accumulation, RIOX1 is also a component of other intranuclear bodies, including clusters of late replicating chromatin, employing a putative role in the establishment or maintenance of some heterochromatic regions [89], besides those in ribosome biogenesis. A RIOX1 function in demethylating lysines 4 and 36 of H3 histone has also been suggested, even if it is still not clear how it can accomplish this function without a known protein domain essential for chromatin interaction [19]. Similarly, a putative regulation of H3K9 methylation by RIOX2 in lung and breast cancer has been reported [23,95]. However, the experimental evidence of such activity is controversial [20], since no demethylation activity was observed in vitro for these putative demethylases towards any methylated histone H3 fragment peptides [96], and the structure of these proteins is not fully compatible with efficient histone demethylase activity [12]. Overall, the prominent role of RIOX1 and RIOX2 proteins as ribosome hydroxylases indicates a role in ribosome maturation, but this remains to be fully understood.

#### 2.2.3. HIF1AN

The hypoxia-inducible factor 1 subunit alpha inhibitor (HIF1AN), also known as FIH1, was identified in 2001 as a protein able to negatively modulate the biological activity of HIF-1 (hypoxia-inducible factor-1) [97,98], the master transcription regulator that leads to the activation of critical adaptive genes during low oxygen stress by recruiting coactivators histone acetyltransferases CBP and p300 through its α-subunit [99]. In normal oxygen conditions, HIF-1 is highly unstable and approaches rapid proteasomal degradation but can escape this destiny in hypoxia [100]. In normoxia, two post-translational modifications negatively regulate HIF-1 stability and activity: the prolyl (P) hydroxylation mediated by the oxygen-dependent prolyl hydroxylases (PHDs) promotes the interaction of HIF-1 with the von Hippel Lindau protein (VHL) E3 ubiquitin-ligase adaptor, favoring its proteasomal degradation; at the same time, the asparagine (N) hydroxylation catalyzed by the oxygen-dependent asparaginyl hydroxylase HIF1AN abrogates the interaction between HIF-1 and its transcriptional coactivators, preventing the expression of hypoxia-induced genes [24,101]. Considering the requirement of dioxygen as a co-substrate for the hydroxylation reactions, HIF1AN may act as an oxygen sensor [25,102], as well as regulate the Notch signal activation pathway [103,104]. However, several other HIF1AN substrates have been recently described outside the HIF pathway (reviewed in [25,105]). Moreover, the enzymatic activity of HIF1AN appears highly promiscuous and not restricted to asparagine hydroxylation [24,25], opening up the possibility of regulating physiological functions still unknown.

#### 2.2.4. JMJD4

JMJD4 was shown to hydroxylate a lysine residue of eukaryotic release factor 1 (eRF1) to improve translational termination efficiency [26] without any known function of histone modification. It was recently reported to interact with Hsp70 to mediate the turnover of PKM2, coding for a pyruvate kinase which is normally absent in healthy adult cardiomyocytes but elevated in cardiomyopathy [27]. According to the evidence, Jmjd4 interacts with Hsp70 and catalyzes the hydroxylation of K66 of PKM2 to promote its degradation through a chaperone-mediated autophagy mechanism.

#### 2.2.5. KDM8 (Alias JMJD5) and JMJD7

JMJD5 and JMJD7 are structurally similar and act as hydroxylases [106] potentially involved in ribosome biogenesis [38] and osteoclastogenesis [28,107]. However, the evidence for arginine hydroxylation has been shown with peptide substrates in vitro without providing in vivo evidence [106]. JMJD5 and JMJD7 have been also identified as proteases that cleave the N-terminal tails of histones H2, H3, and H4 containing methylated arginine or lysine residues (reviewed in [31]), but this remains to be proven. Indeed, despite the presence of a double-strand beta-helix that might remember hydrolytic enzymes, structural insights in their catalytic domain corroborate their function as hydroxylases rather than hydrolases [38]. For JMJD5, it has been proposed there may also a exist demethylase function towards H3K36me2, but this activity was later questioned because of difficulties with experimental in vitro reproducibility [96,106] and due to JMJD5′s catalytic site structure, which is not idoneous to accommodate methylated lysine residues [108,109]. Besides their uncertain catalytic roles, JMJD5 and JMJD7 may have indirect effects on protein stability. It has been recently shown that both wild-type and catalytically inactive mutants of JMJD5 cooperate with E3 ligase HUWE1 to destabilize EGFR and EGFR tyrosine kinase inhibitor-resistant mutants for proteasomal degradation in lung cancer cells [110]. A study reported that JMJD5, despite being mostly a nuclear protein, can shuttle between the cytoplasm and cell nucleus [111]. However, it is still unclear which are the cellular conditions regulating its subcellular localization. Evidence that JMJD5 and JMJD7 may have both pro- and anti-cancer functions is discussed in Oh et al., 2019 [22]. Curiously, a natural readthrough transcription between the JMJD7 locus and the downstream neighbor phospholipase A2, group IVB (PLA2G4B), occurs in tumor tissues, encoding the fusion protein JMJD7-PLA2G4B including a partial JmjC domain and the C2 and phospholipase A2 domains. The incomplete JmjC domain predicts the absence of any enzymatic activity so that the functions of this fusion protein likely match those of phospholipase A2 [22], making it difficult to distinguish the worth of JMJD7′s contribution in such tumor tissues.

#### 2.2.6. JMJD6

JMJD6 is one of the most debated proteins in biology [112,113]. It was originally described as a phosphatidyl-serine receptor [114], and hence first named PSR (phosphatidylserine receptor), then, later identified as a nuclear protein unrelated to this putative function [115,116], but surprisingly able to bind PS [117,118]. To date, JMJD6 has been characterized as demethylase toward histone [32,33] and non-histone arginines [119] or towards methylated ssRNA [33], but also as lysine hydroxylase [34,37,120,121,122,123,124] and is even able to indirectly influence RNA splicing processes [34,36,125,126]. Moreover, in vitro assays suggested the ability of JMJD6 to non-specifically interact with single-strand RNA (ssRNA) [13], but the eventual biological significance of this possible interaction in vivo remains to be proven. Although inconsistent demethylation activity toward histone arginine [120,123] or lysine [35] has also been reported, structural peculiarities of JMJD6 resemble KDM enzymes, suggesting that its function as a demethylase cannot be ruled out [127]. To make the scenario even more confusing, JMJD6 turned out to have proteolytic activity (reviewed in [31]) and a putative new tyrosine kinase function [128].

However, independently of its catalytic activity, JMJD6 may also have a role in the modulation of DNA damage promoting the downregulation of H4K16ac around DSBs through its physical association with SIRT1 [129], as well as in its response to DNA double-strand breaks at rDNA repeats by regulating the recruitment of the nucleolar protein NSB1 upon DNA damage [130]. Because of the disparate, countless, and sometimes conflicting functions attributed to JMJD6 over the years, there is an urgent need to elucidate many outstanding points, including the catalytic activity of this highly debated protein by conducting systematic, comprehensive, and independent investigation counting not only in vitro assays.

#### 2.2.7. JMJD8

JMJD8 is the first JmjC domain-containing protein found in the lumen of the endoplasmic reticulum possibly involved in protein complex assembly and protein folding [131]. JMJD8 acts as a positive regulator of the TNF-induced NF-κB signaling pathway [132] and regulates STING-induced type I IFN responses [133]. Its regulatory role in the NF-kB pathway is related to its demethylation action on trimethylated lysine of AKT1, with a subsequent alteration of its protein function [40]. The protein has also been shown to interact with pyruvate kinase M2 and to play a role in angiogenesis and cellular metabolism [39]. Overall, little is known about JMJD8, and more examinations are required to establish its functions.

#### 2.2.8. TYW5

The tRNA yW-synthesizing enzyme 5 (TYW5) enables several functions, including iron ion binding activity and protein homodimerization activity, but, more importantly, it acts as an RNA hydroxylase. TYW5 is involved in the carbon hydroxylation of wybutosine (yW), a hypermodified guanosine nucleoside that is essential for correct phenylalanine codon translation [41], into hydroxywybutosine (OHyW) in tRNA (Phe) [134]. While the regulatory functions of its action have not been dissected, TYW5 has been recently identified as a schizophrenia risk gene [135]. Again, the biological functions of this protein should be more deeply examined.

### 2.3. Non-Catalytic Functions of Active Histone Demethylases

In this section, we report some relevant biological effects which have been observed when catalytically active KDMs were modulated, and which did not depend on their demethylase activity.

#### 2.3.1. KDM6A (Alias UTX) and KDM6B (Alias JMJD3)

KDM6A and KDM6B are active histone lysine demethylases that specifically act on H3K27me2/3, thus playing a central role in histone code especially in regulating HOX genes and development [56,57,58,136]. KDM6A is an X-linked gene that escapes X-inactivation [137]. KDM6B is an autosomal gene that shares high sequence homology with KDM6A and UTY, despite its lack of the tetracopeptide repeats [58]. Although catalytically active, KDM6A and KDM6B play demethylase-independent roles in the chromatin remodeling of differentiated cells to regulate T-box family members’ expression, mediating the recruitment of SWI–SNF complex [138]. In mouse embryonic stem cells, KDM6A, independently of its enzymatic activity, controls mesoderm differentiation and UTY expression [139]. KDM6A, redundant with UTY, shows high demethylase-independent activity [15,139,140]. Indeed, the overlapping activity of KDM6A and UTY in embryonic development is likely due to H3K27 demethylase-independent mechanisms [15]. The demethylase activity of UTX-1, the *C. elegans* homologous of mammalian KDM6A, was also dispensable for embryonic development and viability, suggesting the presence of important demethylase-independent functions during nematode development [141]. A core intrinsically disordered region (cIDR) of KDM6A has been described as crucial for histone methyltransferase MLL4 recruitment to cell condensates [142,143], shedding light on a possible mechanism underlying the functions besides the enzymatic activity of KDM6A [144]. On the other hand, KDM6B prevents the full reprogramming of MEFs (mouse embryonic fibroblasts) to iPSCs (induced pluripotent stem cells) through a demethylase-independent pathway that involves it in the regulation of PHD finger protein 20 (PHF20) degradation [145]. Like KDM6A and KDM6B, many other chromatin-modifying enzymes retain functions in the absence of their catalytic activity [142].

#### 2.3.2. KDM1A

Lysine-specific histone demethylase 1A (KDM1A, also known as LSD1) demethylates H3K4me1/2 and H3K9me1/me2 in the context of a variety of developmental processes [8]. This functional complexity involves an association with nuclear factors and non-coding RNA which occur both at catalytic and non-catalytic regions of the enzyme [145]. In many regulatory processes, KDM1A operates in strict cooperation with multi-subunit complexes such as CoREST, CtBP, or NuRD [42]. While in these contexts it is very difficult to separate KDM1A’s catalytic and non-catalytic contributions, distinct functions completely independent from demethylase activity have been described. KDM1A destabilizes the F-box protein FBXW7 and abrogates its functions independent of its demethylase activity [146]. This protein acts as a substrate recognition subunit of SCF (SKP1-CUL1-F-box protein) E3 ubiquitin ligase complex and targets human oncoproteins, such as Cyclin E, c-JUN, c-MYC, NOTCH-1, and MCL-1, for ubiquitylation and proteasome degradation [147]. In this regard, FBXW7 functions as a typical tumor suppressor [148]. KDM1A directly binds to FBXW7 and competitively decreases the binding ability of FBXW7 on its bona fide substrates. More importantly, KDM1A reduces the protein level and half-life of FBXW7 [146]. This is also observed in the case of enzymatically dead mutants and cannot be blocked by KDM1A inhibitors [147]. Another case in which KDM1A binds to and destabilizes a protein regards p62, a key component of autophagic machinery that promotes autophagy activation [149]. The final effect of this action in gynecologic cancers is autophagy inhibition. In a different context, the association with KDM1A leads to protein stabilization. This is the case when KDM1A binds to ERRα, the Estrogen-related receptor α, an orphan nuclear receptor without a natural ligand, acting as a transcriptional regulator. KDM1A binding protects it from proteasome-dependent degradation [150]. Recently, new evidence has provided insights into the demethylase-independent roles of KDM1A in regulating cellular differentiation [151]. KDM1A catalytically inactive mutants caused a mild effect on gene expression during mammalian embryogenesis, while KDM1A depletion determined an increase in H3K27ac and p300 acetyl-transferase occupancy at KDM1A-regulated enhancer regions, allowing for a global transcriptional de-repression and impairing cell fate transition [151]. KDM1A also seems to repress the p53 signaling pathway in a demethylase-independent manner to promote prostate cancer reprogramming [152].

#### 2.3.3. KDM1B

Lysine-specific demethylase 2 (KDM1B, also known as LSD2) acts on H3L4me2/3, but it could also perform functions beyond its catalytic activity of demethylase; however, very little is known about this. In S. pombe, both KDM1A and KDM1B are involved in the repression of heterochromatic regions through demethylase-dependent and -independent mechanisms [153]. It has been proposed that the N-terminal Zf-CW domain of KDM1B is essential to exploit demethylase-independent repression functions [154].

#### 2.3.4. KDM2B

Lysine-specific demethylase 2B (KDM2B, also named JHDM1B) demethylates H3K4me3 and H3K36me2, but some authors report that it may also demethylate H3K79 [49]. KDM2B is a component of the Polycomb complex 1 subtype 1 (PRC1.1) and is required for its recruitment to unmethylated CpGs. PRC2 can subsequently be recruited [155,156]. Studies with mESCs show that both PRC1 and PRC2 complexes can interact with KDM2B. However, while the actual recruitment of the PRC1.1 complex to chromatin by KDM2B is not dependent on its demethylase activity, its enzyme activity could be involved in other functions stimulated by these interactions (discussed in [157]).

#### 2.3.5. KDM4A

Lysine-specific demethylase 4A (KDM4A), also termed JMJD2A and JHDM3A, can demethylate histone H3 lysine 9 (H3K9) and 36 (H3K36) and H1.4K26 [8,50,51,158]. KDM4A may also bind in vitro the methylated H3K4 and the H4K20 through its Tudor domains, but the biological consequences of this binding remain to be established [159]. Besides its catalytic functions on chromatin, KDM4A has been shown to have a crucial role in the induction of senescence-associated phenotypes. In a ternary complex, it interacts with the methylated form of p53 and FBX022. This association facilitates ubiquitylation of p53 by SCF complexes. In this case, KDM4A is likely to act as a scaffold independent of its demethylase activity. Downregulation of methylated p53 by SCFFbxo22 is required for the induction of p16 and senescence-associated secretory phenotypes (SASPs), the former of which is critical for permanent cessation of cell proliferation upon any mitogen stimuli [160].

#### 2.3.6. KDM4B

Lysine-specific demethylase 4B (KDM4B), also termed as JMJD2B and JHDM3B, has the same substrate specificity of KDM4A but shows distinct non-catalytic functions. KDM4B is essential for ER-mediated transcription in breast cancer cell lines through direct interaction with ER and with its regulatory targets [161]. While its demethylase activity is required for this function, non-catalytic functions may also be involved. KDM4B has been shown to interact with several transcription factors and members of chromatin modification complexes, including components of the SWI/SNF-B [162] and MLL2 [161] complexes. KDM4B interacts with the transcription factor GATA-3 in breast cancer cell lines and directly co-activates transcription factor activity in reporter-based experiments [163]. It is not clear to what extent KDM4B demethylation of repressive H3K9me3 marks within upstream regulatory regions of the ER gene is required to allow the binding of GATA-3 to drive receptor expression. KDM4B contributes to the formation of an active complex with transactivation function 1 (AF1) and steroid receptor coactivator 3 (SRC-3) in the presence of estrogen [163]. Moreover, KDM4B and ER were shown to form a ternary complex with FBX022 [164]. The amount of this complex plays a key role in cofactor dynamics in ER, as shown by perturbation of its protein turnover [164,165].

#### 2.3.7. KDM5 Demethylases

Jamshidi et al. (2021) [166] characterized a mouse strain expressing a KDM5B splicing variant lacking the ARID domain and five amino acids in the JmjN domain. They showed that, although the protein is catalytically inactive, the mouse is viable and fertile, contrary to the KDM5B knockout mouse, which displays an embryonic lethal phenotype [157]. These data demonstrate that while the loss of KDM5B catalytic function can be surrogated by the other KDM5 demethylases active during development, the protein cannot be substituted in some non-catalytic functions that are essential for normal embryonic development. Many experimental observations support the biological effects of KDM5 demethylases, independently of their catalytic activity [167,168,169,170]. Indeed, KDM5 proteins can also act as transcriptional repressors in a demethylase activity-independent manner by recruiting other repressive complexes [167,168]. For example, KDM5B has been involved in anti-tumor immunity by favoring the SETDB1-dependent repression of retroelements in melanoma [168]. Conversely, KDM5s can sometimes directly support transcription independently of their demethylase activity, functioning as a scaffold for transcription factors in specific cell contexts [171]. For instance, KDM5A in a complex with CLOCK–BMAL can inhibit the activity of the histone deacetylase HDAC1, enhancing histone acetylation and transcriptional activation at the Per2 promoter [169]. 

KDM5A and KDM5B are overexpressed in many cancers and play a role in the response to genomic damage and drug tolerance [5,172]. It has been reported that both proteins contribute to replication stress response and tolerance [170]. First, they positively regulate RRM2, the regulatory subunit of ribonucleotide reductase and a major determinant of replication stress tolerance. Moreover, they are also required for optimal levels of activated Chk1, a major player of the intra-S phase checkpoint that protects cells from replication stress [170]. KDM5A is enriched at ongoing replication forks and associates with both PCNA and Chk1. Therefore, KDM5A/B proteins are required for both RRM2 overexpression and tolerance to hydroxyurea. Altogether, these results indicate that KDM5A/B are major players in the cell response to replication stress. A striking result of the study is that drugs targeting the enzymatic activity of KDM5 proteins do not attenuate the effects of KDM5A/B overexpression [170]. In the context of cancer, there is compelling evidence of direct interactions and close interplay of KDM5A and KDMB with the Retinoblastoma protein RB, and both catalytic and not-catalytic KDM5 functions are involved in this cross-talk (reviewed in [173]). 

A new frontier in the non-catalytic functions of the KDM5 class is represented by their effects on RNA maturation. It was shown that KDM5B can recruit protein complexes involved in the mRNA processing machinery, implying an alternative and not catalytic epigenetic action mediated by this protein in gene regulation [174].

Drosophila KDM5 (Lid) is a direct regulator of genes required for mitochondrial structure and function [175,176]. Significantly, this regulatory function occurs independently of KDM5 histone demethylase activity. Instead, it requires the association to methylated H3K4 mediated by the PHD domain. Genome-wide, KDM5 binding overlaps with the active chromatin mark H3K4me3, and a fly strain specifically lacking H3K4me2/3 binding shows defective KDM5 promoter recruitment and gene activation [175]. KDM5 therefore plays a central role in regulating mitochondrial function by utilizing its ability to recognize specific chromatin contexts. Recent data suggest that KDM5-mediated regulation of mitochondrial activity is also likely to play a key role in humans [177]. A recent screening on Lid interactors shed new light on other potential demethylase-independent activities of KDM5 [178].

## 3. KDM Isoforms without Catalytic Activity

**Figure 1:**
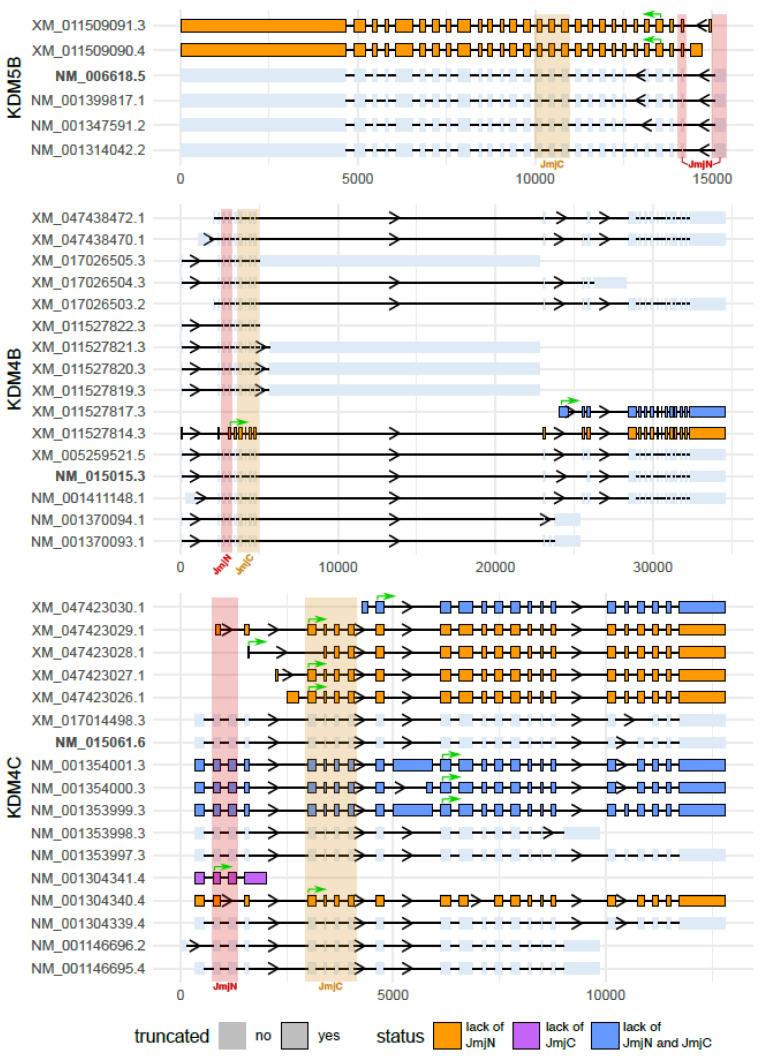
Schematic representation of the predicted and validated transcripts for KDM4 and KDM5 genes (from NCBI Gene RefSeq database). Each of these transcripts could generate different predicted or validated protein isoforms with or without known protein domains, resulting in non-truncated or truncated isoforms. Among the truncated protein isoforms, we highlighted the absence of JmjN, JmjC, or both of these domains, using orange, purple, and blue, respectively. Green arrows show the translational start sites for transcripts giving rise to shorter protein isoforms. For KDM4A, KDM4D, KDM5A, KDM5C, and KDM5D, no truncated protein isoforms were predicted. More details are in Table S1. Figure 1 was created using R v4.3.1 and ggplot2 package v3.5.1. Final editing with Inkscape 1.3.2.

KDM genes generally produce a great variety of transcripts due to multiple transcription initiation sites and/or alternative splicing. These transcripts may potentially or effectively give rise to multiple protein isoforms. Some of these isoforms may show drastically different functional properties. In particular, the KDM4 and KDM5 classes encompass predicted protein isoforms without the amino-terminal part, thus lacking the JmjN domain which, together with the JmjC domain and some residues of the ARID domain, is required for their histone demethylase activity [179,180] (Figure 1). We recently showed that a predicted KDM5B amino-terminal truncated isoform (KDM5B-NTT) is indeed expressed in several cancer cell lines [181]. The transcript giving rise to KDM5B-NTT originates from a secondary transcription start site located downstream of the first ATG and makes use for translation of a downstream ATG in the fourth exon. The resulting protein isoform lacks the JmjN domain and part of the ARID domain [181] and is therefore catalytically inactive [179,180].

**Figure 2:**
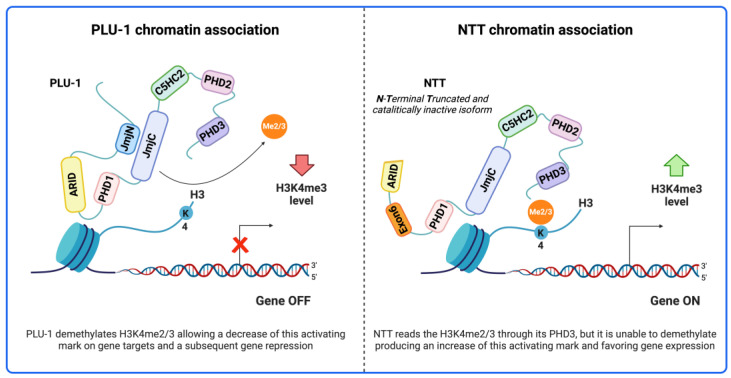
Model of the competitive action of the KDM5B-NTT isoform on the KDM5B-PLU1 targets. Created with BioRender.com.

It also includes an additional exon (exon-6) previously attributed to other putative isoforms [182]. While the KDM5B-NTT transcript is much less abundant than that one giving rise to the canonical PLU-1 isoform, the truncated protein is much more stable to proteasome degradation and can accumulate in breast cancer cells, although its relative amount is variable in different cell lines. We showed that KDM5B-NTT over-expression in breast cancer cell lines leads to a significant increase in H3K4 tri-methylation of bulk chromatin and transcriptional induction of several genes, including genes previously found directly regulated by the canonical PLU-1 isoform. Therefore, KDM5B-NTT may compete with the PLU-1 isoform acting as a negative modulator [181]. This is somewhat expected, since the truncated isoform is inactive but retains the PHD domains and is potentially able to address it to target chromatin loci [54,180] (Figure 2). Besides these antagonistic effects, KDM5B-NTT could retain some of the non-catalytical functions of PLU-1, starting with those determined by protein–protein interactions which are based on domains that are shared with PLU-1. N-truncated predicted isoforms also exist in the KDM4 class (Figure 1) where JmjN is also required for catalytic activity, so it will be very relevant to test their effective expression.

The expression of inactive demethylase isoforms corroborates the existence of functions not strikingly linked to histone demethylase activity and might help truly distinguish between direct and indirect actions on chromatin modifications.

## 4. Conclusions and Perspectives

**Figure 3:**
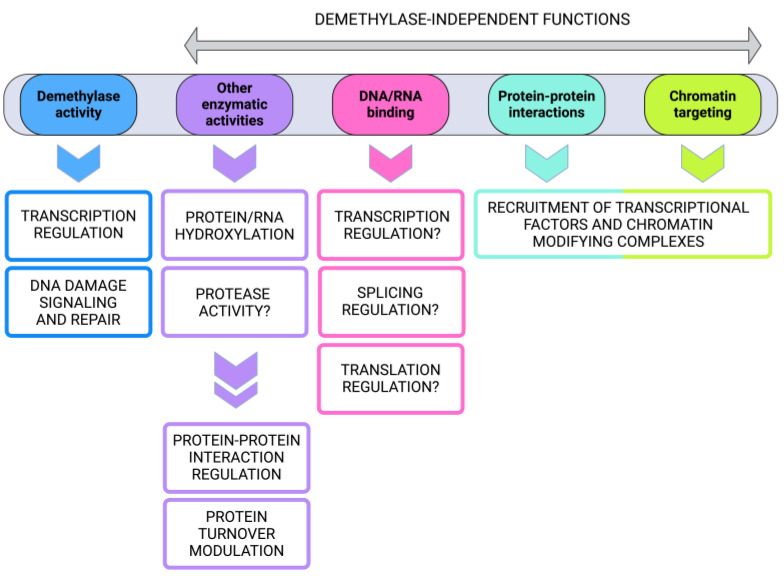
Schematic representation of different protein domains that confer demethylase activity or determine demethylase-independent functions, including other enzymatic activities, which in turn regulate many biological processes. Created with Biorender.com.

After twenty years of research, the superfamily of histone lysine demethylases appears as an extremely versatile group of proteins that expanded their potential by acquiring novel catalytic activities and non-catalytic functions (Figure 3).

This has been possible thanks to the complexity of their catalytic cores and their richness in structural modules capable of interacting with other proteins and nucleic acids’ structural motifs. While most of the effort of the scientific community has been focused on the catalytic functions of the various classes and the effects of their impairment by mutations and chemical inhibitors, individual members and peculiar isoforms have shown biological effects independent from demethylase activity. This is a very relevant aspect to be considered for a true understanding of the biological role of these proteins. We think that in the future more energy should be invested in distinguishing between the catalytic and non-catalytic effects of this fascinating group of proteins.

## Figures and Tables

**Table 1:** Table of KDM proteins with described demethylase-independent functions and/or non-canonical catalytic functions. The description of the principal protein domains is shown in the inset legend. Created with BioRender.com.

**Jumonji Histone Demethylases with No or Uncertain Histone Demethylase Activity**						
**Official Symbol (Synonyms)**	**Protein Name**	**Uniprot Entry**	**Protein Domains**	**Substrates**	**Catalytic** **Activity**	**Refs**
**JARID2 ** (JMJ, DIDDF7)	Protein Jumonji	Q92833	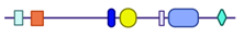	-	None	[8,14]
**UTY ** (KDM6C, KDM6AL)	Ubiquitously transcribed tetratricopeptide repeat containing, Y-linked	O14607	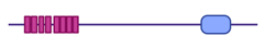	H3K27me2/3	Demethylase (uncertain)	[15,16,17]
**JmjC-Only Domain Proteins with Different Catalytic Functions**						
**Official Symbol (Synonyms)**	**Protein Name**	**Uniprot Entry**	**Protein Domains**	**Substrates**	**Catalytic** **Activity**	**Refs**
**HSPBAP1** (PASS1)	HSPB1-associated protein 1	Q96EW2	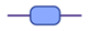	HSP27	Not investigated	[18]
**RIOX1 ** (MAPJD, NO66, C14orf169, JMJD9)	Ribosomal oxygenase 1	Q9H6W3	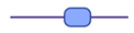	RPL8, H3K4me1/2/3, H3K36me2/3	Hydroxylase, Demethylase (uncertain)	[12,19,20,21,22]
**RIOX2 ** (MINA53, NO52, JMJD10, MINA, MDIG)	Ribosomal oxygenase 2	Q8IUF8	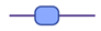	RPL27A, H3K9me3	Hydroxylase, Demethylase (uncertain)	[12,20,21,22,23]
**HIF1AN ** (FIH)	Hypoxia-inducible factor 1 subunit alpha inhibitor	Q9NWT6	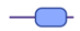	HIF1-2α, self hydroxylation at Trp-296, ankyrin repeat domain (ARD)-containing proteins	Hydroxylase	[24,25]
**JMJD4**	Jumonji domain-containing 4	Q9H9V9	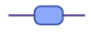	eRF1, PKM2	Hydroxylase	[26,27]
**KDM8 ** (JMJD5)	Lysine demethylase 8	Q8N371	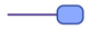	H3K9me1, HEK36me2, NFATc1	Hydroxylase, **Demethylase?** Protease?	[28,29,30,31]
**JMJD6** (KIAA0585, PTDSR1, PTDSR, PSR)	Jumonji domain-containing 6	Q6NYC1	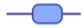	Arginines, lysines H3R2me, H4R3me, 7SK, MePCE, p53, GLS RNA	Hydroxylase, Demethylase (uncertain), Protease?	[31,32,33,34,35,36,37]
**JMJD7**	Jumonji domain-containing 7	P0C870	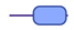	H3R2me2, H4R3me2, DRG1/2	Hydroxylase, Protease?	[31,38]
**JMJD8 ** (C16orf20, PP14397)	Jumonji domain-containing 8	Q96S16	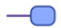	AKT1	Demethylase	[39,40]
**TYW5 ** (C2orf60, HTYW5)	tRNA wybutosine-synthesizing protein 5	A2RUC4	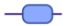	yW-72 in tRNA(Phe)	Hydroxylase	[41]
**Catalytically Active KDMs with Catalytic-Independent Functions**						
**Official Symbol (Synonyms)**	**Protein Name**	**Uniprot Entry**	**Protein Domains**	**Substrates**	**Catalytic** **Activity**	**Refs**
**KDM1A ** (LSD1, AOF2, BHC110, CPRF, KDM1, KIAA0601)	Lysine-specific histone demethylase 1A	O60341	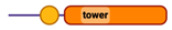	H3K4me1/2 H3K9me1/2	Demethylase	[8,42]
**KDM1B ** (LSD2, AOF1, C6orf193)	Lysine-specific histone demethylase 2	Q8NB78	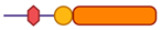	H3K4me1/2	Demethylase	[43,44,45]
**KDM2B ** (CXXC2, FBL10, FBXL10, JHDM1B, NDY1, PCCX2)	Lysine-specific demethylase 2B	Q8NHM5	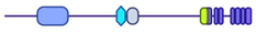	H3K4me3 H3K36me2 H3K79me2/3	Demethylase	[46,47,48,49]
**KDM4A** (JHDM3A, JMJD2, JMJD2A, KIAA0677, TDRD14A)	Lysine-specific demethylase 4A	O75164	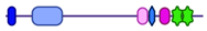	H3K9me3 H3K36me3 H1.4K26	Demethylase	[8,50,51]
**KDM4B** (JHDM3B, JMJD2B, KIAA0876, MRD65, TDRD14B)	Lysine-specific demethylase 4B	O94953	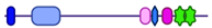	H3K9me3	Demethylase	[52]
**KDM5A ** (RBP2, RBBP2, JARID1A)	Lysine-specific demethylase 5A	P29375	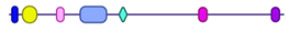	H3K4me2/3	Demethylase	[53]
**KDM5B ** (PLU-1, CT31, RBBP2H1A, PPP1R98, JARID1B)	Lysine-specific demethylase 5B	Q9UGL1	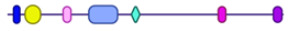	H3K4me2/3	Demethylase	[54,55]
**KDM6A ** (UTX)	Lysine-specific demethylase 6A	O15550	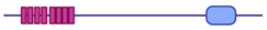	H3K27me2/3	Demethylase	[56,57,58]
**KDM6B ** (JMJD3, KIAA0346)	Lysine-specific demethylase 6B	O15054	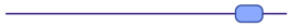	H3K27me2/3	Demethylase	[56,57,58,59]
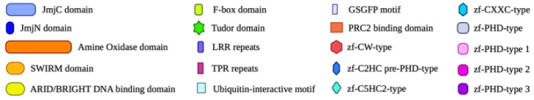						

## Data Availability

No new data were created or analyzed in this study. Data sharing is not applicable to this article.

## Supplementary Materials

The following supporting information can be downloaded at: https://www.mdpi.com/article/10.3390/ijms25136900/s1, reference citation [183].
